# Evaluating Soluble Solids in White Strawberries: A Comparative Analysis of Vis-NIR and NIR Spectroscopy

**DOI:** 10.3390/foods13142274

**Published:** 2024-07-19

**Authors:** Hayato Seki, Haruko Murakami, Te Ma, Satoru Tsuchikawa, Tetsuya Inagaki

**Affiliations:** 1 Institute of Agricultural Machinery, National Agricultural and Food Research Organization, 1-40-2, Nisshin-Cho, Kita-Ku, Saitama City 331-8537, Japan; sekih179@affrc.go.jp; 2 Graduate School of Bioagricultural Sciences, Nagoya University, Furo-Cho, Chikusa, Nagoya 464-8601, Japanmate@agr.nagoya-u.ac.jp (T.M.); st3842@agr.nagoya-u.ac.jp (S.T.)

**Keywords:** white strawberry, Vis-NIR spectroscopy, non-destructive, spectral noise, fruit pigment, Brix

## Abstract

In recent years, due to breeding improvements, strawberries with low anthocyanin content and a white rind are now available, and they are highly valued in the market. Strawberries with white skin color do not turn red when ripe, making it difficult to judge ripeness. The soluble solids content (SSC) is an indicator of fruit quality and is closely related to ripeness. In this study, visible–near-infrared (Vis-NIR) spectroscopy and near-infrared (NIR) spectroscopy are used for non-destructive evaluation of the SSC. Vis-NIR (500–978 nm) and NIR (908–1676 nm) data collected from 180 samples of “Tochigi iW1 go” white strawberries and 150 samples of “Tochigi i27 go” red strawberries are investigated. The white strawberry SSC model developed by partial least squares regression (PLSR) in Vis-NIR had a determination coefficient *R*^2^_p_ of 0.89 and a root mean square error prediction (RMSEP) of 0.40%; the model developed in NIR showed satisfactory estimation accuracy with an *R*^2^_p_ of 0.85 and an RMSEP of 0.43%. These estimation accuracies were comparable to the results of the red strawberry model. Absorption derived from anthocyanin and chlorophyll pigments in white strawberries was observed in the Vis-NIR region. In addition, a dataset consisting of red and white strawberries can be used to predict the pigment-independent SSC. These results contribute to the development of methods for a rapid fruit sorting system and the development of an on-site ripeness determination system.

## 1. Introduction

Strawberries, renowned globally for their aesthetic appeal, superior sensory attributes, and robust nutritional profile, continue to be the focus of breeding efforts worldwide, which aim to cultivate novel and distinct varieties. Among these, the white strawberry, a variant diverging from the traditional red strawberry, has been developed and has gained considerable popularity in Japan. The market for white strawberries could expand to the international market as a luxury fruit and gift because of their unusual appearance, good aroma, and sweetness. The white strawberry was registered as a variety in 2009, and breeding has been underway since then. The characteristic red hue in strawberries is attributed to anthocyanins, primarily Pelargonidin 3-Glucoside and Pelargonidin 3-Rutinoside. Notably, the concentration of these anthocyanins in white strawberries is markedly lower in comparison to their red counterparts [1,2].

In the commercial market, strawberries are selected based on external features such as vibrant red coloration, size, form, and the absence of surface imperfections. Nevertheless, it is the internal quality attributes, including sweetness, acidity, and aroma, that significantly sway consumer preferences [1,3]. Sweetness, a critical determinant of flavor, is predominantly quantified by the soluble solids content (SSC), which is conventionally measured using a refractometer and denoted in degrees Brix. The SSC can be used as a valuable indicator of fruit maturity because fruits accumulate sugar as they mature [4]. This method, however, is invasive and labor-intensive. The challenge with white strawberries lies in their minimal color transformation upon ripening, which poses difficulties in manual ripeness assessment [1,2]. Current ripeness indicators for strawberry timing are based solely on fruit surface color. The sorting of strawberry fruit is based on external attributes such as color distribution, fruit size and shape, and the absence of physiological abnormalities, and is primarily a visual inspection [5].

Visible–near-infrared (Vis-NIR) spectroscopy and near-infrared (NIR) spectroscopy, which are extensively utilized in food analysis, offer a non-destructive and rapid method of evaluation. The near-infrared spectral range (800–2500 nm) is characterized by broad, overlapping bands arising from fundamental vibrations of molecular bonds (C-H, O-H, N-H), along with harmonics and combination tones [6]. This technique has been employed to assess various physiological properties of strawberries, such as firmness, SSC, pH, titratable acidity, and total phenolic content [5,7]. Developing sorting technology that can non-destructively sort white strawberries by external and internal qualities will increase consumer satisfaction, confidence, competitiveness, and profitability. For the non-destructive evaluation of white strawberries, SSC estimation by near-infrared hyperspectral imaging with the application of NIR has been used [8], but there is no research using NIR. Since hyperspectral imaging equipment is expensive and difficult to use in practice, the relatively inexpensive Vis-NIR or NIR spectroscopy is easier to introduce into a sorting system.

Visible near-infrared spectroscopy, encompassing the visible spectrum (380–800 nm), is also used to evaluate parameters such as color, titratable acidity, SSC, and total phenolic content [1,9]. Predictive models for numerous quality attributes of strawberries have been developed using this approach. Vis-NIR has color information that influences the model. Evaluating strawberries with a white pericarp in Vis-NIR can contribute to the discussion of the influence of color information on the model and improve its robustness.

In previous approaches, improving accuracy has been considered by optimizing chemometrics; in actual applications, optimizing measurement conditions on the instrument side is required. Since measurement errors in absorbance affect quantitative analysis, evaluating the S/N of each measurement condition of the spectrophotometer is adequate. This information is also helpful for instrument design.

The objective of this research is to ascertain the efficacy of Vis-NIR and NIR spectroscopy in measuring the SSC of white strawberries. To achieve this, the SSC estimation of white strawberries was performed from Vis-NIR and NIR spectra based on chemometric methods. In the same way, an SSC estimation model was constructed for red strawberries and compared with the SSC estimation model for white strawberries. In addition, it is necessary to optimize the measurement conditions for practical application development. Since noise caused by measurement conditions affects model accuracy, we examined methods to evaluate noise.

## 2. Materials and Methods

### 2.1. Fruits Materials

**Figure 1:**
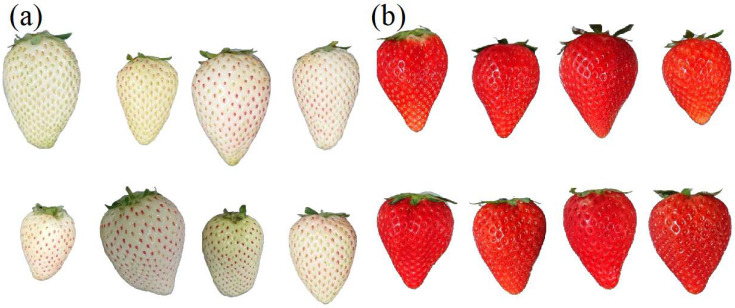
(**a**) White strawberry “Tochigi iW1 go”. (**b**) Red strawberry “Tochigi i27 go”.

Strawberry samples of “Tochigi iW1 go” with white skin (Figure 1a) were obtained from the Strawberry Research Institute-Tochigi Prefectural Agricultural Experiment Station (Tochigi-shi, Tochigi Pref. 328-0007, Japan) between February and March 2021. Hereafter, we refer to “Tochigi iW1 go” as white strawberries. “Tochigi i27 go with a red skin (Figure 1b)” were grown in Sano City, Tochigi Prefecture, and were purchased in March 2021. Hereafter, we refer to “Tochigi i27 go” as red strawberries. We sampled 180 white strawberries and 150 red strawberries. Judging from appearances, there were no extremely underripe samples of white or red strawberries, and both ripeness and shape were generally considered within the range of ripeness available in the marketplace. Prior to the experiment, the strawberries were stored under controlled conditions at 23 °C to minimize variation in the measurements due to temperature changes. The samples were refrigerated after harvest and transported; they were kept in the refrigerator for approximately one hour prior to measurements. No serious visual quality deterioration was observed during the experiment.

### 2.2. Spectral Measurements

The absorbance spectra of the white and red strawberries were measured using a spectrophotometer with a Vis-NIR wavelength range and a spectrophotometer with a NIR wavelength range. The whole fruit was measured, but the measurement points were limited to the fruit equator, where measurements could be taken stably. These systems are both compact and lightweight and can be carried around and measured on site.

The Vis-NIR spectrophotometer (Fruits Selector, Kubota Co., Osaka, Japan) measurement wavelength range is 500–978 nm, with a wavelength resolution of 2 nm. To obtain sufficient light intensity, the number of integrations was set to 16 and the exposure time was set to 150 ms for the white strawberries and 75 or 100 ms for the red strawberries. The light source was halogen (35 W), and the white plate and dark current were measured inside the device.

The NIR spectrophotometer (MicroNIR1700, JDSU Co., Milpitas, CA, USA) measurement wavelength range is 908–1676 nm, with a wavelength resolution of 6.19 nm. To obtain sufficient light intensity, the number of integrations was set to 128 and the exposure time was set to 7.4 ms for the white strawberries and red strawberries. The light source was two vacuum tungsten lamps (<1 W each lamp) placed near the detector. The white plate was measured as a reference with the light source on. The dark current was measured with the lights off.

The average absorbance was calculated using Equation (1) by measuring four points at the equators of the red and white strawberries, approximately every 60 degrees, using each device.

(1) $$A_{\lambda }=-log\frac{S_{\lambda }-D_{\lambda }}{W_{\lambda }-D_{\lambda }}$$

where *λ* represents wavelength, $A_{\lambda ,t}$ represents absorbance at wavelength *λ*, *S* and *W* represent sample intensity and white reference intensity, respectively, and D denotes dark intensity.

### 2.3. Measurement of Soluble Solid Content

After collecting the spectral data, the Brix value as the SSC of each sample was measured using a Brix meter (PAL-1, ATAGO Co., Ltd., Tokyo, Japan) from whole fruit. The juice obtained by squeezing the fruit in a non-woven wrapper was measured.

### 2.4. Data Analysis

#### 2.4.1. Dataset

Six datasets consisting of Vis-NIR and NIR spectra of the white strawberries, red strawberries, and a mixture of white and red strawberries, as well as the SSC of each sample, were created. It is practical to apply the calibration model to multiple strawberry varieties, including red and white strawberries. Obvious outlier spectra were excluded from the analysis. Each dataset was split at a 7:3 ratio into a training set for model building and a test set for validation.

#### 2.4.2. Spectral Pre-Processing

Second derivative processing was applied to each spectrum using the Savitzky–Golay algorithm (window size: 15) to remove noise, for baseline correction, and to reduce band overlap.

#### 2.4.3. PLSR Modeling

The SSC estimation models were constructed from the training data using partial least squares regression (PLSR) analysis. The prediction error sum of squares (PRESS) values were calculated from the predicted values for each sample using leave-one-out cross-validation, as shown in Equation (2). PLS factors (The latent variable for the PLSR model) were determined by the F statistic to ensure that the PRESS was not significantly larger than the minimum PRESS, since adopting the number of factors that yield the minimum PRESS may result in overfitting [10].

(2) $$PRESS=\sum_{i=1}^{n}{\left(y_{i}-\hat{y_{i}}\right)}^{2}$$

where $y_{i}$ represents the measured SSC values for each sample, and $\hat{y_{i}}$ represents the predicted value in cross-validation.

#### 2.4.4. Validation

Each constructed model was applied to the test data to verify model performance. The coefficient of determination of the cross-validation, calibration, and prediction (*R*^2^cv, *R*^2^c, and *R*^2^p); the root mean square error (RMSE) of the cross-validation, calibration, and prediction (RMSECV, RMSEC and RMSEP); and the ratio of performance to deviation (RPD) were calculated as indicators to evaluate the model. A higher coefficient of determination is desirable and a lower RMSE is better for the model. An RPD value below 1.5 indicates that the model cannot be used to describe the dataset, while values between 1.5 and 2.0 indicate that the model is able to discriminate between low and high values of the response variable adequately; values between 2 and 2.5 indicate that approximate quantitative predictions are possible. For values between 2.5 and 3.0 or above 3.0, the prediction is classified as good or excellent, respectively [11,12,13]. These indices were calculated using Equations (3)–(5). The overall reliability of the model should be judged from these indicators, and there should be no deviation from the results of the cross-validation, calibration, and prediction.

(3) $$RMSECV,RMSEC,RMSEP=\sqrt{\frac{1}{n}\sum_{i=1}^{n}{\left({\hat{y}}_{i}-\bar{y}\right)}^{2}}$$

(4) $${R^{2}}_{cv}\;,{{R^{2}}_{c}\;,{\;R}^{2}}_{p}=1-\left\{\frac{\sum_{i=1}^{n}{\left(y_{i}-\hat{y}\right)}^{2}}{\sum_{i=1}^{n}{\left(y_{i}-\bar{y}\right)}^{2}}\right\}$$

(5) $$RPD=\frac{\sigma_{y\_test}}{RMSEP}$$

where *n* represents the number of samples; *y* represents the SSC values measured using the Brix meter; $\bar{y}$ denotes the mean values of *y*; and $\hat{y}$ denotes the Brix value forecast using Vis-NIR or NIR spectroscopy during cross-validation, calibration, and prediction; $\sigma_{y\_test}$ represents the standard deviation of the SSC for the test sets.

### 2.5. Strawberry Pigment

In strawberries, the primary pigments are anthocyanin, which is a red pigment, and chlorophyll, which is a green pigment. To explore their absorption wavelengths, sample solutions were prepared. This research utilized Pelargonidin 3-Glucoside, a prominent anthocyanin in strawberries. The Pelargonidin 3-Glucoside powder (Toronto Research Chemicals, North York, ON, Canada) was solubilized in water to a concentration of 0.1 (mg/mL). Similarly, chlorophyll samples were created by dissolving chlorophyll powder (Tokyo Chemical Industry Co., Ltd., Tokyo, Japan) in water to a concentration of 0.2 (mg/mL). Spectrophotometric analysis was conducted using a UV-Vis-NIR spectrophotometer (UV-3100PC, SHIMADZU Co., Kyoto, Japan), with spectra gathered in the 190–3100 nm range at 1 nm resolution. The 450–800 nm range was selected for analysis. The intensities of the pigment sample ($I_{t}$) and the reference water ($I_{0}$) were measured in a quartz cell. The absorbance of the pigments was calculated using the following Equation (6).

(6) $$A_{\lambda }=-log\left(\frac{I_{0,\lambda }}{I_{t,\lambda }}\right)$$

where *λ* represents wavelength, $A_{\lambda }$ represents absorbance at wavelength *λ*, and $I_{0}$ and $I_{t}$ represent water intensity and pigment sample intensity at the wavelength, respectively.

### 2.6. Noise Evaluation of Spectrometer

Errors in quantitative analysis are important for the absorbance measurement errors, i.e., the instrumental errors. These noise evaluations are also useful for new equipment design. The Vis-NIR spectrophotometer and NIR spectrophotometer used in this study allow the exposure and number of integrations to be set. The reference was measured repeatedly by varying the exposure and integration to evaluate the potential noise effects due to measurement conditions. In the case of the Vis-NIR spectrophotometer, 30 conditions were set with 6 exposure times (10, 60, 120, 180, 240, and 300 ms) and 5 integration times (8, 16, 32, 64, and 128 times); in the case of the NIR spectrophotometer, there were 6 exposure times (0.01, 1.5, 3.0, 4.5, 6.0, and 7.4 ms), and the number of integrations was set to 30 conditions with 5 steps (8, 16, 32, 64, and 128 integrations). The white plates were measured 20 times each under these conditions. The apparent absorbance was calculated according to Equation (7). Nineteen apparent absorbance spectra were calculated for each condition based on the first measurement.

(7) $$A_{\lambda ,t}=-log\frac{W_{\lambda ,t}-D_{\lambda ,t}}{W_{\lambda ,t0}-D_{\lambda ,t0}}$$

where *λ* represents wavelength, $A_{\lambda ,t}$ represents absorbance at wavelength *λ*, $W_{\lambda ,t0}$ represent white reference intensity at the first measurement and after the first measurement, respectively, and *D* denotes dark intensity.

The obtained spectra were converted to second derivative spectra using the Savitzky–Golay algorithm. The standard deviations of the second derivative of absorbance for each of the 20 points in the wavelength ranged from 834 nm to 872 nm for Vis-NIR and from 1435 nm to 1552 nm for NIR and were calculated using Equation (8). These wavelength bands are also the range where high-intensity luminance values are measured in the obtained luminance spectra. This calculation yields the standard deviation spectrum (for 20 wavelengths) for each exposure and the number of integrations. Lastly, for the noise level (NL), the average value of the 20 wavelengths of the standard deviation spectrum obtained for each exposure condition and integrations were calculated using Equation (9).

(8) $$\sigma_{\lambda }=\sqrt{\frac{\sum_{i=1}^{N}{\left(\left(\ddot{A_{\lambda ,i}}\right)-\bar{\ddot{A_{\lambda }}}\right)}^{2}}{N-1}}$$

where *λ* represents wavelength, $\sigma_{\lambda }$ represents the standard deviation of the samples at each wavelength point, $\ddot{A_{\lambda ,i}}$ represents the second derivative of absorbance at the wavelength, $\bar{\ddot{A_{\lambda }}}$ represents the mean second derivative of absorbance at the wavelength, and *N* represents degrees of freedom: sample size.

(9) $$NL=\frac{\sum \sigma_{\lambda }}{M}$$

where *NL* represents noise level, and *M* represents degrees of freedom: number of wavelengths selected.

## 3. Results and Discussion

### 3.1. Sugar Content Distribution

**Figure 2:**
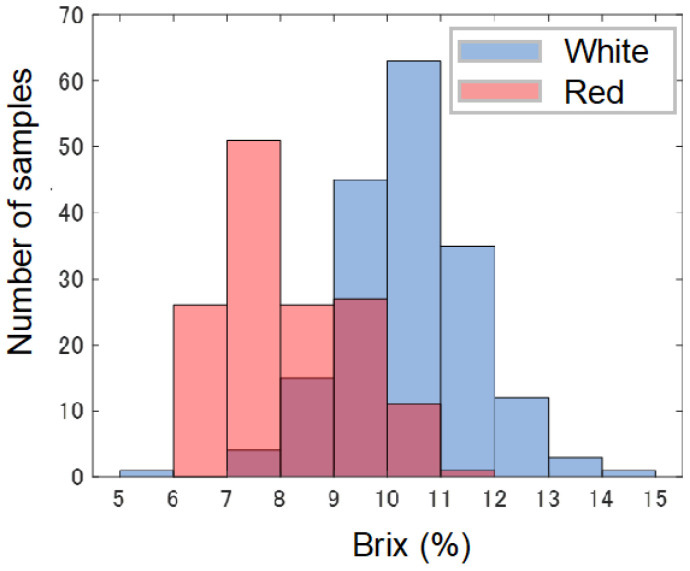
The distribution of white and red strawberry Brix reference values.

Figure 2 shows histograms of the SSC for the white and red strawberries. The mean and standard deviation of the SSC for the white strawberries were 10.04% and 1.29, respectively. On the other hand, the mean and standard deviation of the SSC for the red strawberries were 8.13% and 1.18%, respectively. Each distribution was determined to be close to a normal distribution. The white strawberries had a higher mean SSC than the red strawberries. The SSCs were widely distributed and likely contained samples of various maturity levels.

### 3.2. Vis-NIR Spectra

**Figure 3:**
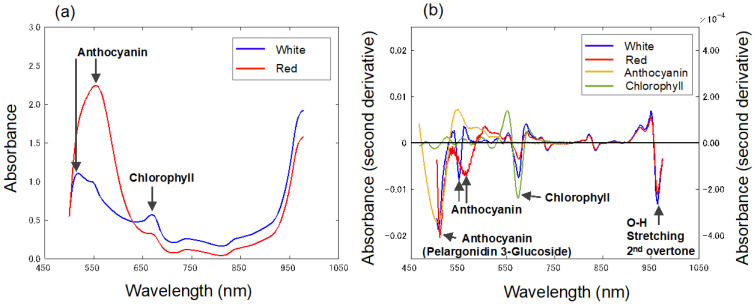
(**a**) Average absorbance spectra of white and red strawberries in the Vis-NIR range. (**b**) Average second derivative absorbance spectra of white and red strawberries and spectra of pigments in strawberries in the Vis-NIR range.

Figure 3a shows the average absorbance spectra of the white and red strawberries measured by the Vis-NIR spectrophotometer. Figure 3b shows the second derivative spectra of the white and red strawberries, anthocyanin, and chlorophyll. The second derivative spectrum of pelargonidin 3-glucoside measured as anthocyanin showed an absorption peak near 505 nm. The white and red strawberries also showed a peak around 505 nm, due to pelargonidin 3-glucoside. The absorption peak near 550 nm in the white strawberries and the absorption peak near 560 nm in the red strawberries are due to a shift in the absorption peak of the anthocyanins appearing near 500–535 nm [14]. It has been noted that anthocyanins in strawberries are less stable because they are affected by vitamin C and amino acids [15]. The absorption peak around 673 nm that appears in the second derivative spectrum of chlorophyll appeared in both the red and white strawberries. The absorption spectrum of the white strawberries shows that they contain anthocyanins and chlorophyll like the red strawberries. It is inferred that white strawberries appear white due to the higher content of chlorophyll and the lower content of anthocyanins, which affect scattering. Red strawberries have lower absorbance at wavelengths longer than 600 nm; so, white strawberries are less reflective in this band. In addition, no differences in the characteristics of the pericarp surface other than color were visually observed; so, it was inferred that the effect of reflection was more limited than the scattering. To verify this, it is necessary to investigate the scattering and absorption coefficients at different wavelengths by microscopic observation of the pericarp surface and Monte Carlo simulation [16,17]. Absorptions near 838 nm and 970 nm associated with sugar-related O-H and C-H [18] appeared in the secondary differential spectra of the red and white strawberries.

### 3.3. NIR Spectra

**Figure 4:**
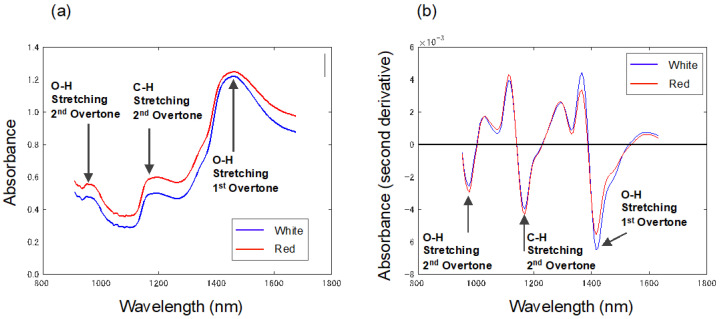
(**a**) Average absorbance spectra of white and red strawberries in the NIR range. (**b**) Average second derivative absorbance spectra of white and red strawberries in the NIR range.

Figure 4a shows the average absorbance spectra of the white and red strawberries measured by the NIR spectrophotometer. Figure 4b shows the second derivative spectra of the white and red strawberries.

**Figure 5:**
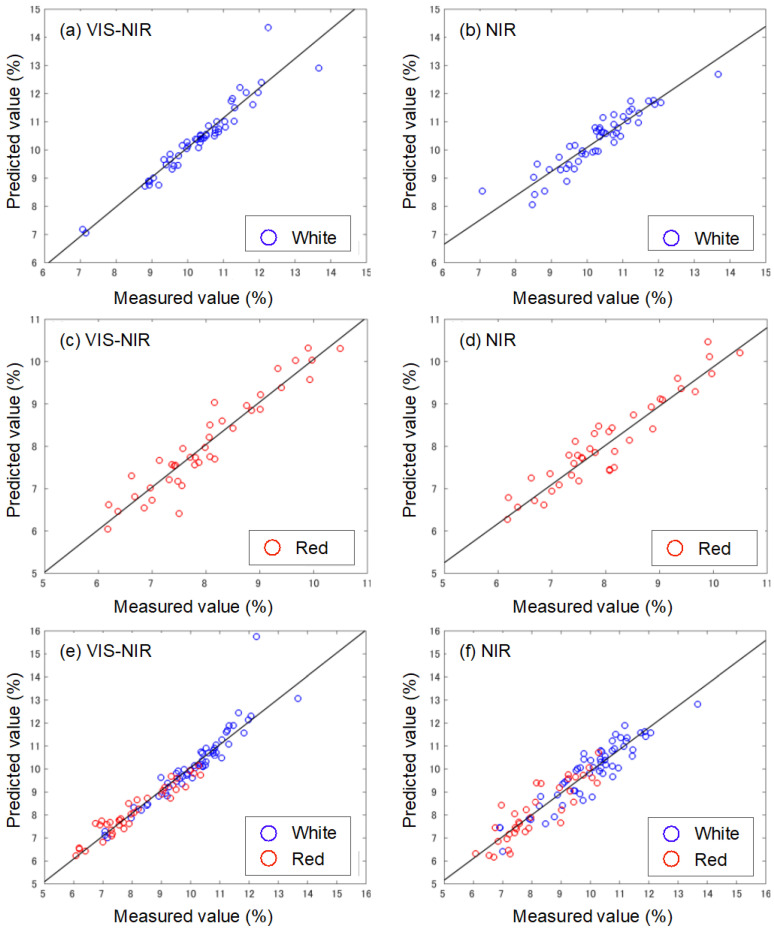
Measured values of Brix and predicted values of Brix obtained by the PLS regression model. Samples used for modeling are (**a**,**b**) for white strawberry, (**c**,**d**) for red strawberry, and (**e**,**f**) for a model created by mixing white and red strawberries. The wavelength range is Vis-NIR for (**a**,**c**,**e**) and NIR for (**b**,**d**,**f**).

In the NIR region, minimal differences were noted in the spectral properties relating to the skin color of strawberries, as compared to the Vis-NIR spectra. The main peaks observed in Figure 5a, at 952, 1187, and 1459 nm, are attributed to the overtones and combination tones of O-H and C-H bonds. Additionally, the peaks at 976, 1075, and 1416 nm in Figure 5b correlate with the O-H bonds of water, while the peak at 1168 nm is associated with the C-H bonds in sugars [18,19].

### 3.4. Determination of Sugar Content

**Figure 6:**
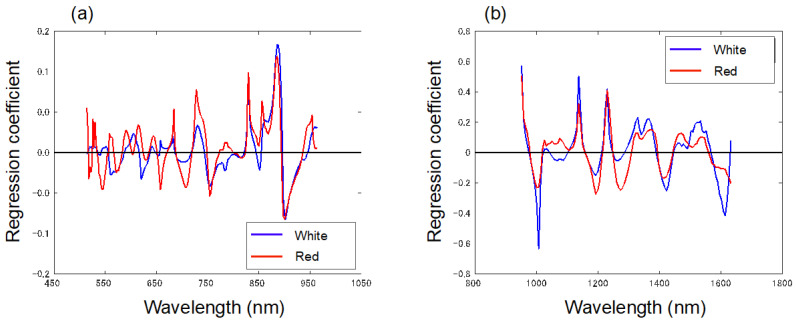
Regression coefficient spectra of white and red strawberries in the range of (**a**) Vis-NIR, (**b**) NIR.

Table 1 shows the PLSR results for the development of the SSC estimation model for each dataset, and Figure 6 shows the relationship between the measured and estimated values for the test set. Since there were no *R***^2^** or RMSE differences between the cross-validation on the training set and the calibration and validation on the test set for all the datasets, we conclude that there is no overfitting in the developed SSC estimation model. The *R*^2^_p_ and RMSEP for the white strawberry SSC estimation model developed in the Vis-NIR wavelength range (Figure 5a) were 0.89 and 0.40%, respectively, while the model developed in the NIR range (Figure 5b) had an *R*^2^_p_ of 0.85 and an RMSEP of 0.43%. Both models were robust with RPD values of 2.98 in the Vis-NIR region and 2.64 in the NIR region. It was shown that SSC estimation was possible with practical accuracy for the white strawberries using the Vis-NIR wavelength range and the NIR wavelength range. The *R*^2^_p_ and RMSEP for the red strawberry SSC estimation model developed in the Vis-NIR wavelength range (Figure 5c) were 0.89 and 0.36%, respectively, while the model developed in the NIR range (Figure 5d) had an *R*^2^_p_ of 0.89 and an RMSEP of 0.36%. Both models were robust with RPD values of 3.05 in the Vis-NIR region and 3.04 in the NIR region. These results indicate that Vis-NIR and NIR can estimate the SSC for strawberries with white skin color as accurately as for red strawberries. The *R*^2^_p_ and RMSEP for the red and white strawberry SSC estimation model developed in the Vis-NIR wavelength range (Figure 5e) were 0.91 and 0.48%, respectively, while the model developed in the NIR range (Figure 5d) had an *R*^2^_p_ of 0.87 and an RMSEP of 0.57%. Both models were robust with RPD values of 3.35 in the Vis-NIR region and 2.27 in the NIR region. Practical models among multiple varieties can be developed with excellent predictive performance, primarily when variability and heterogeneity are well represented in the calibration set. A comparison of this model [20,21,22] with other fruit multiple-variety models showed similarly good estimation accuracy. The results suggest that it is possible to construct a practical estimation model for the SSC estimation from a dataset of strawberry varieties with different peel colors in both the Vis-NIR and NIR regions.

### 3.5. Model Replacement

To further examine the influence of skin color on SSC estimation in Vis-NIR and NIR, we attempted to estimate the SSC for the white strawberries with a model built from a red strawberry dataset and the SSC for red strawberries with a model built from a white strawberry dataset. Table 2 shows the results of the application of the SSC estimation model to data from the different color test sets. The *R*^2^_p_ and RMSEP of the red strawberry SSC estimation model developed in the Vis-NIR wavelength range applied to the white strawberry test data were 0.80 and 1.03%, respectively. The *R*^2^_p_ and RMSEP of the white strawberry SSC estimation model developed in the Vis-NIR wavelength range applied to the red strawberry test data were 0.09 and 6.55%, respectively. This indicates that the pigments affect the SSC estimation in the Vis-NIR region. Figure 6a shows the regression coefficients of the SSC estimation model developed from the red strawberry and white strawberry datasets in Vis-NIR. The position and size of the peaks of the regression coefficients were different below 800 nm, including pigments such as anthocyanins and chlorophyll. However, the model developed from the dataset consisting of the red and white strawberries could estimate the SSC. These results suggest that sufficient sampling of the red and white strawberry data could reduce the influence of pigment on the model. On the other hand, the *R*^2^_p_ and RMSEP of the red strawberry SSC estimation model developed in the NIR wavelength range applied to the white strawberry test data were 0.65 and 0.60%, respectively. The *R*^2^_p_ and RMSEP of the white strawberry SSC estimation model developed in the NIR wavelength range applied to the red strawberry test data were 0.73 and 0.76%, respectively. Since pigment absorption information does not appear in the near-infrared region, applying the model to test sets with different skin colors did not seriously decrease estimation accuracy. The positions of the peak wavelengths of the regression coefficients in the NIR region for the red and white strawberries were generally consistent, suggesting that the difference in peak size affected the accuracy of the estimation.

### 3.6. Noise Evaluation of Spectrometer

**Figure 7:**
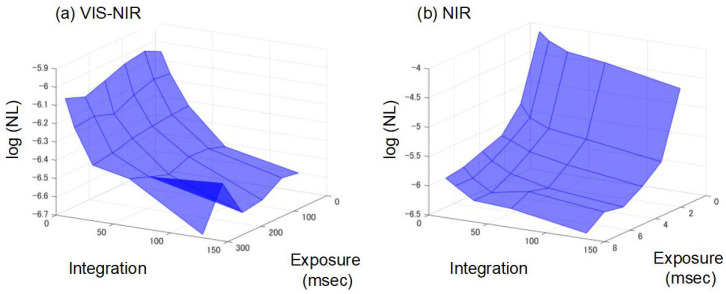
Relationship between exposure time, number of integrations, and noise level (NL) of (**a**) Vis-NIR spectrometer, (**b**) NIR spectrometer.

Figure 7 shows the relationship between noise level, number of integrations, and exposure time for the white plates measured with the Vis-NIR spectrophotometer (Figure 3a) and the NIR spectrophotometer (Figure 3b). For the set range of integration times and exposure times, the noise level of the Vis-NIR spectrophotometer is lower when the number of integrations increases than when the exposure time increases. On the other hand, for the NIR spectrophotometer, increasing the exposure time decreases the noise level more than increasing the number of integrations. Furthermore, the NIR spectrophotometer shows a larger decrease in noise level under different conditions than the Vis-NIR spectrophotometer. The light source of the Vis-NIR spectrophotometer is more intense and has a longer exposure time than the light source of the NIR spectrophotometer, which improves the stability of the measurement. The minimum noise level was 0.208 μabs for the Vis-NIR spectrophotometer with an exposure time of 300 ms and 128 integrations; for the NIR spectrophotometer, the noise level was 0.331 μabs with an exposure time of 7.4 ms and 128 integrations. The measurement conditions for the strawberries when using the NIR spectrophotometer in this study were minimal noise levels. In the Vis-NIR spectrophotometer, the noise level can be reduced more by increasing the number of integrations, but this should be considered in conjunction with other factors, such as the practicality of the measurement speed. Thus, it was shown that it is effective to consider the measurement conditions according to the noise level. The relationship between noise level and SSC estimation performance in such basic experiments is also helpful for determining the specifications of new practical devices when they are designed.

## 4. Conclusions

This study explored the possibility of Vis-NIR spectroscopy and NIR spectroscopy as valid methods for the non-destructive SSC evaluation of white strawberries. The SSC estimation model was developed by PLSR using the second derivative spectrum. The results showed good estimation accuracy with an R^2^_p_ of 0.89 and RMSEP of 0.40% for Vis-NIR and an R^2^_p_ of 0.85 and RMSEP of 0.43% for NIR. These estimation accuracies were comparable to the results of the model for red strawberries. Vis-NIR spectroscopy and NIR spectroscopy are effective methods for the SSC of strawberries regardless of the color of the skin. The relationship between the non-destructive measurements and sensory evaluation results for each strawberry fruit can be investigated to verify whether the SSC estimation by Vis-NIR and NIR for strawberries is useful for ripeness and quality evaluation. As with the red strawberries, the second derivative spectra in the Vis-NIR region observed an absorption derived from anthocyanin and chlorophyll pigments in the white strawberries. When estimating the SSC for red and white strawberries in a single model in the Vis-NIR region, biased sampling may cause the model to become unstable.

Furthermore, these results contribute to the development of methods for a rapid fruit sorting system and the development of an on-site ripeness determination system. For practical development, it is desirable to optimize for low noise within acceptable measurement conditions (speed, light source intensity, etc.) using a noise level index. The noise level indicator used in this study was able to visualize differences in spectral noise for each measurement condition. The results of this research will contribute to the development of a practical white strawberry quality evaluation system.

## Figures and Tables

**Table 1:** Characteristics of the PLS regression models for the prediction of Brix in the white and red strawberries based on the spectra of fruits.

Model				Calibration	Cross-Validation		Prediction					
Strawberry Color	Wavelength	PLS Factor	*R* ^2^_C_	RMSEC (%)	*R* ^2^_CV_	RMSECV (%)	*R* ^2^_p_	RMSEP (%)	RPD_p_	Slope	Offset	Bias
White	Vis-NIR	7	0.96	0.26	0.95	0.30	0.89	0.40	2.98	1.06	−0.54	0.07
White	NIR	6	0.84	0.53	0.80	0.59	0.85	0.43	2.64	0.82	1.92	0.06
Red	Vis-NIR	7	0.92	0.34	0.87	0.44	0.89	0.36	3.05	1.01	−0.01	0.04
Red	NIR	7	0.88	0.42	0.84	0.48	0.89	0.36	3.04	0.91	0.80	0.07
White and Red	Vis-NIR	8	0.96	0.34	0.95	0.38	0.91	0.48	3.35	0.99	0.14	0.08
White and Red	NIR	7	0.91	0.52	0.89	0.56	0.87	0.57	2.77	0.93	0.60	−0.07

**Table 2:** Characteristics of the PLS regression models for the prediction of Brix in the white and red strawberry based on the spectra of fruits.

Sample	Model	Wavelength	*R* ^2^_p_	RMSEP (%)	RPD_p_	Slope	Offset	Bias
White	Red	Vis-NIR	0.80	1.03	1.25	1.05	−1.30	0.78
Red	White	Vis-NIR	0.09	6.55	0.18	0.40	11.22	−6.35
White	Red	NIR	0.65	1.15	1.11	0.60	3.24	0.86
Red	White	NIR	0.73	1.16	1.02	0.76	2.93	−0.99

## Data Availability

The original contributions presented in the study are included in the article, further inquiries can be directed to the corresponding author.

## References

[1] da Silva F.L., Escribano-Bailón M.T., Pérez Alonso J.J., Rivas-Gonzalo J.C., Santos-Buelga C. (2007). Anthocyanin pigments in strawberry. LWT—Food Sci. Technol..

[2] Tsurumi R., Nakanishi T., Ishihara Y., Ohashi T., Kojima N., Saitou Y., Kobayashi Y., Hatakeyama A., Iimura K., Handa T (2020). Breeding of a New Strawberry Cultivar with White Fruits ‘Tochigi i W1 go’. Bull. Tochigi Prefect. Agric. Exp. Stn..

[3] Di Vittori L., Mazzoni L., Battino M., Mezzetti B. (2018). Pre-harvest factors influencing the quality of berries. Sci. Hortic..

[4] Wilson D. (2021). Chemical Sensors for Farm-to-Table Monitoring of Fruit Quality. Sensors.

[5] Sánchez M.-T., De la Haba M.J., Benítez-López M., Fernández-Novales J., Garrido-Varo A., Pérez-Marín D. (2012). Non-destructive characterization and quality control of intact strawberries based on NIR spectral data. J. Food Eng..

[6] Ozaki Y., Huck C., Tsuchikawa S., Engelsen S.B. (2021). Near-Infrared Spectroscopy: Theory, Spectral Analysis, Instrumentation, and Applications.

[7] Amodio M.L., Ceglie F., Chaudhry M.M.A., Piazzolla F., Colelli G. (2017). Potential of NIR spectroscopy for predicting internal quality and discriminating among strawberry fruits from different production systems. Postharvest Biol. Technol..

[8] Seki H., Ma T., Murakami H., Tsuchikawa S., Inagaki T. (2023). Visualization of Sugar Content Distribution of White Strawberry by Near-Infrared Hyperspectral Imaging. Foods.

[9] Saad A., Azam M.M., Amer B.M.A. (2022). Quality Analysis Prediction and Discriminating Strawberry Maturity with a Hand-held Vis–NIR Spectrometer. Food Anal. Methods.

[10] Haaland D.M., Thomas E.V. (1988). Partial least-squares methods for spectral analyses. 1. Relation to other quantitative calibration methods and the extraction of qualitative information. Anal. Chem..

[11] Zhao N., Wu Z., Zhang Q., Shi X., Ma Q., Qiao Y. (2015). Optimization of Parameter Selection for Partial Least Squares Model Development. Sci. Rep..

[12] Gowen A.A., Downey G., Esquerre C., O’Donnell C.P. (2011). Preventing over-fitting in PLS calibration models of near-infrared (NIR) spectroscopy data using regression coefficients. J. Chemom..

[13] Ferrara G., Marcotuli V., Didonna A., Stellacci A.M., Palasciano M., Mazzeo A. (2022). Ripeness Prediction in Table Grape Cultivars by Using a Portable NIR Device. Horticulturae.

[14] Kitsuda K., Irie M., Nakamura T., Inno Y., Nishioka T., Tsuji H. (2003). Estimation of Nasunin Content in the Skin of Eggplant ‘Mizunasu’ Fruits by Nondestructive and Rapid Method. Nippon Shokuhin Kagaku Kogaku Kaishi.

[15] Aamer R.A., Amin W.A., Attia R.S. (2021). Enhancement of color stability in strawberry nectar during storage. Ann. Agric. Sci..

[16] Zhang M., Li C., Yang F. (2019). Optical properties of blueberry flesh and skin and Monte Carlo multi-layered simulation of light interaction with fruit tissues. Postharvest Biol. Technol..

[17] Ma T., Li X., Inagaki T., Yang H., Tsuchikawa S. (2018). Noncontact evaluation of soluble solids content in apples by near-infrared hyperspectral imaging. J. Food Eng..

[18] Golic M., Walsh K., Lawson P. (2003). Short-Wavelength Near-Infrared Spectra of Sucrose, Glucose, and Fructose with Respect to Sugar Concentration and Temperature. Appl. Spectrosc..

[19] Włodarska K., Szulc J., Khmelinskii I., Sikorska E. (2019). Non-destructive determination of strawberry fruit and juice quality parameters using ultraviolet, visible, and near-infrared spectroscopy. J. Sci. Food Agric..

[20] Yu Y., Yao M. (2023). Is this pear sweeter than this apple? A universal SSC model for fruits with similar physiochemical properties. Biosyst. Eng..

[21] Wang J., Wang J., Chen Z., Han D. (2017). Development of multi-cultivar models for predicting the soluble solid content and firmness of European pear (*Pyrus communis* L.) using portable vis–NIR spectroscopy. Postharvest Biol. Technol..

[22] Wang Z., Wu S., Zuo C., Jiang M., Song J., Ding F., Tu K., Lan W., Pan L. (2024). Exploring the variability and heterogeneity of apple firmness using visible and near-infrared hyperspectral imaging. LWT.

